# NK cell tolerance as the final endorsement of prenatal tolerance after *in utero* hematopoietic cellular transplantation

**DOI:** 10.3389/fphar.2015.00051

**Published:** 2015-03-18

**Authors:** Amir M. Alhajjat, Amanda E. Lee, Beverly S. Strong, Aimen F. Shaaban

**Affiliations:** ^1^Department of Surgery, University of Iowa, Iowa City, IAUSA; ^2^Center for Fetal Cellular and Molecular Therapy and The Department of Surgery, Cincinnati Children’s Hospital Medical Center, Cincinnati, OHUSA

**Keywords:** *In utero* transplantation, T cells, NK cells, fetus, tolerance, chimerism

## Abstract

The primary benefits of *in utero* hematopoietic cellular transplantation (IUHCT) arise from transplanting curative cells prior to the immunologic maturation of the fetus. However, this approach has been routinely successful only in the treatment of congenital immunodeficiency diseases that include an inherent NK cell deficiency despite the existence of normal maternal immunity in either setting. These observations raise the possibility that fetal NK cells function as an early barrier to allogeneic IUHCT. Herein, we summarize the findings of previous studies of prenatal NK cell allospecific tolerance in mice and in humans. Cumulatively, this new information reveals the complexity of the fetal immune response in the setting of rejection or tolerance and illustrates the role for fetal NK cells in the final endorsement of allospecific prenatal tolerance.

*In utero* hematopoietic cellular transplantation (IUHCT) remains a promising intervention for treatment of a wide variety of congenital disease (Merianos et al., 2008). A primary assumption in IUHCT is that the early-gestation fetus has an immature immune system that is incapable of rejecting a donor cell transplant. As a result, the introduction of donor antigen prior to the development of the adaptive immunity should lead to life-long donor-specific tolerance. Thus, current protocols for IUHCT favor that the initial transplant be delivered by 12 weeks gestation within a “therapeutic window” that opens shortly after prenatal diagnosis and closes with thymic maturation (Westgren, 2006). Observations of naturally occurring hematopoietic chimeras demonstrates that, in essence, this is feasible (Owen, 1945). However, repeated clinical failure of IUHCT in the setting of a non-defective immune system has forced a re-examination of this central dogma, i.e., the translation of bedside observations back to the bench for hypothesis-driven inquiry.

## The Clinical Paradigm for Prenatal Transplantation

Two related observations arising from clinical experience with IUHCT are in need of a scientific explanation. First, clinical application of IUHCT has documented success in the treatment of severe combined immunodeficiency (SCID). Indeed, the greatest clinical success has been realized in the treatment of NK cell deficient SCID (xSCID or ADA-SCID) whereas the use of IUHCT for the treatment of congenital diseases in which the fetal immune system is not defective has been uniformly unsuccessful (Touraine et al., 1992; Flake et al., 1996; Wengler et al., 1996) This includes most of the clinical experience with IUHCT for hemoglobinopathies such as sickle cell disease or thalassemia. Second, the maternal immune system has been intact for every case despite the nature of the clinical outcome (success or failure) suggesting no independent role for the maternal immune response in IUHCT-related engraftment failures. Taken together, these observations frame the clinical paradigm for IUHCT and serve as a template for translational study.

It has been postulated that competition between the donor and recipient cells for a limited number of available host hematopoietic stem cell (HSC) niches is responsible for the clinical failure of IUHCT in the treatment of hemoglobinopathy (Peranteau et al., 2004, 2006). Favorable competition with the host cells for available niches within the fetal liver or bone marrow is vital for successful engraftment and likely explains the enhanced clinical and experimental success of IUHCT with the use of more competitive fetal donor cells or larger doses of bone marrow cells (Shaaban and Flake, 1999; Peranteau et al., 2006; Shaaban et al., 2006). Improved competition for available host niches would logically lead to higher levels of early chimerism. Previous reports from our group illustrate that the early chimerism level (discussed below) is the major determinant of successful allogeneic engraftment and link this to the development of donor-specific NK cell tolerance (Shaaban et al., 2006; Durkin et al., 2008a,b; Alhajjat et al., 2013). However, a competitive-niche model struggles to explain the dichotomous observations for immunodeficient vs. non-immunodeficient cases and seems to disregard the obvious difference. More specifically, no direct evidence exists to support the existence of quantitative differences in the number of HSCs or available stem cell niches between the xSCID and sickle cell disease or β-thalassemia patients. To the contrary, the defects in SCID emerge following the lineage-specific differentiation of HSCs rather than during their maintenance or self-renewal (reviewed in Schmalstieg and Goldman, 2002; Kalman et al., 2004) As a result, the pre-thymic SCID fetal hematopoietic microenvironment should theoretically have the same frequency of available stem cell niches as in pre-thymic fetus with defective β-globin production and should engraft similarly if niche availability is the limiting factor. Therefore, a model in which donor cell competition for host niches solely determines the outcome of IUHCT does not adequately reconcile the clinical paradigm and prompts further study of early gestation fetal alloimmunity.

Studies in murine and primate models of IUHCT support the presence of an early gestation immune barrier to allotransplantation (Peranteau et al., 2007; Durkin et al., 2008a,b). In a murine study by Peranteau et al. (2007), both congenic and allogeneic transplant recipients demonstrated similar multi-lineage engraftment at 1 week of age. Thereafter, most allogeneic recipients lost engraftment (Peranteau et al., 2007). Similarly, we have also demonstrated that congenic recipients maintain long-term engraftment regardless of the chimerism level whereas allogeneic recipients require a minimum level of circulating chimerism to maintain stable engraftment and prevent a chronic form of rejection (Durkin et al., 2008b). Lastly, despite promising results in other large animal models (Lee et al., 2005; Vrecenak et al., 2014), numerous studies of allogeneic IUHCT in non-human primates have yield poor overall engraftment regardless of the gestational age (Cowan et al., 1996; Shields et al., 2003, 2004). In general, the use of fetal conditioning or mature T cell co-transplantation resulted in higher engraftment rates and chimerism levels overall (Peranteau et al., 2002; Hayashi et al., 2004; Ashizuka et al., 2006). Collectively, these findings point to the existence of a previously unrecognized immune barrier to IUHCT that resides within the fetal host potentially complicating the kinetics of early engraftment.

## Fetal T Cells are Unlikely to Act Alone in Rejection after IUHCT

In the complexity of the developing fetus, successful prenatal engraftment likely requires that all components of the immune system develop tolerance. Similarly, failed engraftment in clinical cases that do not involve immunodeficiency likely results from a lack of tolerance in one or more components of the fetal immune response. In either case, an intrinsic immune barrier to prenatal allotransplantation exists within the fetal host and awaits further delineation. In the greater context, a better understanding of the critical parameters regulating the emergence of self-tolerance may explain the pattern of success and failure of IUHCT.

Previous studies reveal that T cell self-recognition is established before birth in most strains of mice including the C57BL6/J and Balb/c inbred strains. The first measurable indicator of self-recognition is the emergence of phenotypically mature T cells as early as E17 (Crispe et al., 1986). Phenotypically mature single-positive T cells have been found in the human thymus as early as 12 weeks gestation (Stites and Pavia, 1979; Haynes et al., 1989). Confirmation of the functional capacity to reject an allograft comes from transplant studies in newborn mice which do not accept fully allogeneic hematopoietic grafts without myeloablation or immunological preparation (Soper et al., 2003). Conversely, the capacity to reject allogeneic grafts does not exist earlier in gestation. The studies by Kim et al. (1998, 1999) first demonstrated that allospecific T cell tolerance can be reliably achieved by IUHCT to the murine fetus at E14 resulting in the deletion and anergy of donor-reactive T cells. Subsequent studies confirmed the allo-receptivity of C57BL/6 and Balb/c fetuses at E14 (Shaaban et al., 1999, 2006; Hayashi et al., 2002;Durkin et al., 2008a,b; Alhajjat et al., 2013; Nijagal et al., 2013). Collectively, these findings suggest that the potential for donor-specific tolerance to reliably develop following IUHCT is lost shortly after the appearance of mature single positive T cells.

Furthermore, in the studies by Kim et al. (1998, 1999), T cell and skin allograft tolerance could be seen with extremely low levels of *microchimerism* (<0.1%) following IUHCT between MHC class I-matched or mismatched strain combinations. These findings seem to conflict with subsequent studies (Ashizuka et al., 2006; Durkin et al., 2008b) demonstrating that tolerance to a hematopoietic graft tolerance requires *macrochimerism* (1–2%) but may be reconciled through an understanding of the different measures of tolerance. Skin-graft acceptance has been shown to reflect T cell rather than NK cell tolerance whereas tolerance to a hematopoietic graft reflects both. NK cells fail to reject allogeneic skin grafts in the absence of IL-15 activation (Kroemer et al., 2008). Thus in sub-threshold microchimeric mice, host T cells may be tolerant to allogeneic skin grafts while host NK cells are not tolerant to allogeneic hematopoietic cells. Additionally, although NK maturation occurs in the early second trimester of human gestation, it continues for several weeks after birth in the B6 mouse (Dorfman and Raulet, 1998). In the aforementioned studies by Kim et al. (1998, 1999), the majority of skin grafts were placed on the microchimeric mice during the prolonged phase of chronic rejection that is typical of low-level prenatal chimerism. As such some degree of hyporesponsiveness to skin and possibly hematopoietic grafts likely exists during this rejection period. Comparative measurements of the allospecific response between T cells and NK cells in microchimeric or sub-threshold chimeric mice might reveal these differences. Thus, the existence of hematopoietic microchimerism during that period appears to be sufficient for donor-specific T cell and skin allograft tolerance to develop but insufficient for NK cell tolerance. As such, the potential for either T cell tolerance or rejection to develop following IUHCT seems to hinge on the timing of transplant rather than the level of chimerism. Perhaps these differences arise from the relatively high-affinity interactions between T cell receptors and peptide-MHC complexes (TCR-pMHC) that regulate much of thymic selection (reviewed in Moran and Hogquist, 2012; Morris and Allen, 2012). Given the high cell dose per kg of fetal body mass previously used for clinical IUHCT, it is likely that sufficient chimerism levels were present for the induction of T cell tolerance. As such, T cell-mediated rejection seems to be an inadequate stand-alone explanation for the failed engraftment seen in the treatment of hemoglobinopathy by IUHCT.

## Fetal NK Cells as an Intrinsic Barrier to Prenatal Allotransplantation and a Target for Immunotherapy

**FIGURE 1:**
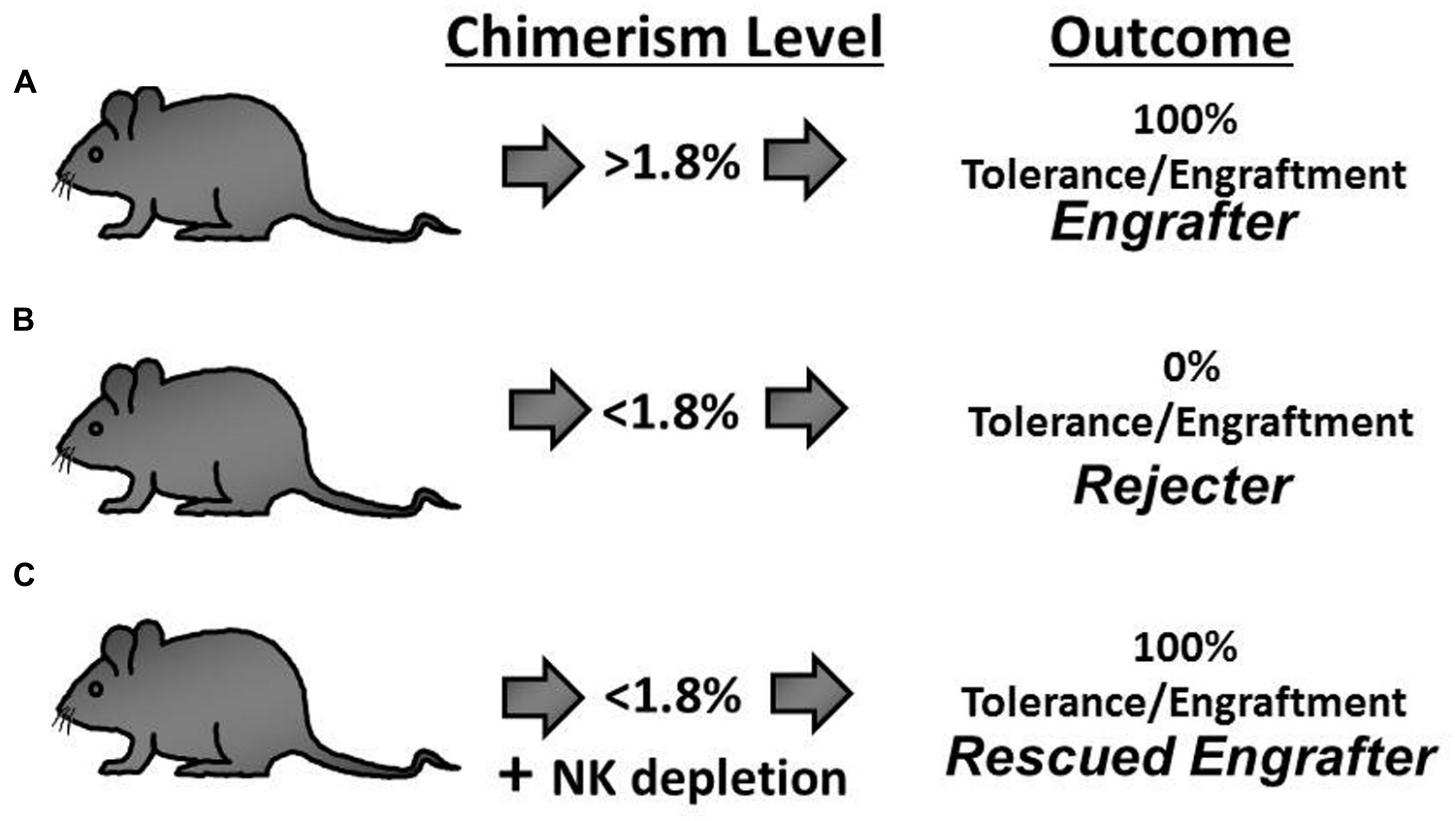
**The outcome of engraftment or rejection following allogeneic *In utero* hematopoietic cellular transplantation (IUHCT) is predicted by the early chimerism level and hinges on the development of NK cell tolerance. (A)** Mice with an early chimerism level above the chimerism threshold (Engrafter mice; >1.8% circulating chimerism) exhibit durable long-term multi-lineage engraftment. **(B)** With early chimerism levels below the chimerism threshold, NK cell tolerance does not develop and universal rejection occurs (Rejecter mice; <1.8% circulating chimerism). **(C)** Rejection is abrogated when NK cells are depleted in rejecter mice thereby rescuing prenatal engraftment (Rescued Engrafter mice).

The presence of a secondary barrier to prenatal engraftment mediated by fetal NK cells would explain the clinical pattern of enhanced success in NK cell deficient recipients (Flake et al., 1996; Wengler et al., 1996; Archer et al., 1997). An NK cell barrier would also explain the delayed experimental engraftment loss (chronic rejection) previously reported by multiple investigators (Carrier et al., 2000; Donahue et al., 2001; Peranteau et al., 2007; Durkin et al., 2008b) as this coincides temporally with the maturation of NK cell allorecognition (Roth et al., 2000). For these reasons, we queried the response of NK cells in a murine model and ultimately confirmed the existence of an NK cell-mediated barrier to the engraftment of prenatally transplanted allogeneic hematopoietic cells (**Figure 1**). Importantly, this barrier was found to be critically dependent on the level of circulating chimerism (Durkin et al., 2008b). With high levels of chimerism, recipients maintained stable engraftment and exhibited donor-specific NK cell tolerance. Conversely, recipients with low chimerism levels displayed NK cell-dependent chronic graft rejection.

The essence of this *quantitative* model for NK cell education is that the donor-specific tolerance requires a threshold level of exposure to the donor ligands during development – a chimerism threshold (>1.8%). Remarkably, we found that the chimerism threshold reliably predicts the binary outcome of either engraftment or rejection which is the arguably most meaningful measure of prenatal tolerance induction. Furthermore, the chimerism threshold proved to be irrelevant in IUHCT between congenic strains of mice which are immunologically matched reducing the likelihood that limiting-dilution kinetics were the cause of failed engraftment below the chimerism threshold (Durkin et al., 2008b). Lastly, we demonstrated that the engraftment loss can be prevented in sub-threshold chimeras by early *in vivo* depletion of host NK cells (**Figure 1**). During the period of NK cell depletion, chimerism levels remained stable or increased slightly. When the host NK cells were allowed to return following withdrawal of the depleting antibody, abrupt rejection was observed in all of the animals. These findings defined a critical relationship between a threshold level of donor chimerism and the development of donor-specific NK cell tolerance. In a larger context, the significance of this threshold lies in the identification of the minimum level of antigen exposure during the T and NK cell education that is necessary for durable recognition as self.

## Trogocytosis as a Mechanistic Link between the Chimerism Threshold and Prenatal NK Cell Tolerance

In order for prenatal tolerance to develop at such low levels of chimerism (1.8%), the process of self-education should include a mechanism to compensate for the low probability of effector cells to encounter appreciable levels of donor ligand. We have recently reported that donor-to-host MHC transfer (trogocytosis) might function in this role (Alhajjat et al., 2013). Trogocytosis of donor MHC may permit sustained *cis*-recognition of the donor ligands during development in the absence of *trans*-interaction directly with the donor cells or with host APC’s. The *cis*-recognition of donor antigens by tolerant host NK cells may selectively prevent the apoptosis of “friendly” phenotypes during the selection of the mature NK cell repertoire. Support for this mechanism comes from the recent report by Brodin et al. (2012) who examined Ly49D+ NK cells in H-2D^d^ transgenic B6 mice and found that the co-expression of Ly49A conferred a resistance to apoptosis. Consistent with this postulate, high levels of trogocytosis were found on the surface of phenotypically friendly NK cells that express donor-specific inhibitory receptors when compared to the phenotypically hostile NK cells that do not express these inhibitory receptors. Subsequent to their developmental selection, the *cis*-recognition of transferred donor-MHC by phenotypically friendly NK cells might provide a continuous exposure to the donor ligands affecting their maturation, survival and function (Kim et al., 2005; Chalifour et al., 2009). In this manner, trogocytosis of donor ligands could provide an intrinsic mechanism for the development and maintenance of donor-specific NK cell tolerance.

## Fetal NK Cells at the Interface with the Maternal Immune System

A maternal immune response toward the donor cells has been proposed as a barrier to prenatal allo-transplantation resulting in the delayed chronic rejection of the donor graft (Merianos et al., 2009; Nijagal et al., 2011). As discussed earlier, this postulate is inconsistent with the recurring clinical observation that IUHCT has been successful in cases where the maternal immune response was intact but the fetal immune system was defective (Flake et al., 1996; Wengler et al., 1996; Merianos et al., 2008). Also incompatible with this conclusion is the observation that engraftment or rejection after IUHCT occurs in littermates subjected to the same maternal influence (Durkin et al., 2008b; Merianos et al., 2009). Lastly, the *in vivo* elimination of host NK cells prevents the chronic rejection seen in sub-threshold chimeras and establishes that the effectors of this response reside within the fetal recipient. Collectively, these findings argue against the existence of a clinically relevant maternal immune barrier to IUHCT and should be reconciled with those of previous reports concluding that a maternal immune response toward the transplanted cells leads to engraftment loss in allogeneic IUHCT.

In the study by Merianos et al. (2009), prenatal allogeneic chimeras were noted to lose engraftment at a relatively high rate several weeks after birth. When naïve foster dams were used after delivery, no engraftment loss occurred. This observation was explained by the finding of donor-specific alloantibodies in the maternal breast milk that perhaps induced graft rejection after birth. The authors provided no data regarding the level of chimerism in the animals that rejected their graft. Additionally, the presence of maternal alloantibodies did not result in graft rejection in nearly 1/3 of the littermates of the pups that lost engraftment suggesting some heterogeneity in the effect of the maternal immune response.

These findings may relate to the experimental methodology employed in their study. The study by Merianos et al. (2009) used very large doses of adult donor bone marrow cells (20 × 10^6^cells/fetus) which provided a large number of mature T cells to the fetus (approximately 10^9^ mature T cells/kg). These large T cell doses may have resulted in a significant graft-vs-host reaction that may have diminished the maternal–fetal immune barrier leading to a greater exposure of the fetal cells to the maternal immune system. The high chimerism levels seen in this model likely compounded the exposure to the donor alloantigens leading to a functionally significant maternal immune response and precipitous fetal loss. Conversely, mature T cells are absent from the fetal liver donor cell populations used in other studies and chimerism levels are much more modest at the lower doses that were used (Rebel et al., 1996; Szilvassy et al., 2001; Taylor et al., 2002; Hayashi et al., 2003). Further study is necessary to confirm the potential to provoke a maternal humoral response with the use of large T cell doses or high early chimerism levels in IUHCT.

These possibilities are further supported by the study of Nijagal et al. (2011), which utilized fetal liver cells for prenatal transplantation and found that the use of naïve foster dams had no impact on delayed engraftment loss. Instead, the authors observed that a significant numbers of T cells traffic from the mother into the fetus following prenatal transplantation and proposed that a maternal T cell response was responsible for the lower early engraftment rates in allogeneic vs. congenic IUHCT. Indeed, higher engraftment rates were seen with the use of T cell deficient mothers or by matching the donor cells with the maternal MHC antigens in wild-type matings thereby avoiding the potential for a maternal T cell response in either setting.

However, the reported early engraftment rate in this study is unusually low given the relatively large number of transplanted fetal liver cells. Using the same methodology to prepare and prenatally transplant a similar dose of MHC mismatched fetal liver donor cells (2 × 10^6^ cells/fetus) in this strain combination, we have reliably detected engraftment in 100% of the offspring despite the use of immunologically normal wild-type dams (Durkin et al., 2008b). The reasons for the different engraftment rates between the two studies are unclear but may result from differences in the strain combination or technical variations in the transplant procedure. Additionally, the authors concluded that maternal T cells persisted in the chimeric offspring for months after birth and led to chronic rejection. However, no maternal cells could be found within the recipient at any point beyond the fetal period making it difficult to reconcile engraftment loss by this mechanism that occurred months later. Despite these unresolved issues, the finding of maternal cells in the fetal immune system illustrates the potential complexities involved in prenatal transplantation and clearly warrants further study.

## Early Gestation Human Fetal NK Cells Possess the Capacity for Allorecognition and the Potential to Respond to Prenatal Allo-Transplantation

The gestational time frame in which human fetal NK cells develop the capacity for allorecognition has not been directly elucidated. The finding of mature cytotoxic NK cells in human cord blood provides clear evidence that this occurs prior to birth (Wang et al., 2007). It is conceivable that this coincides with acquisition of killer immunoglobulin-like receptors (KIR) similar to the acquisition of the homologous Ly49 NK cell receptors in mice (Roth et al., 2000). Acquisition of Ly49 receptors is commensurate with development of mature cytotoxic capacity and temporally coincides with rejection in sub-threshold chimeras or tolerance and engraftment in above threshold chimeras (Anfossi et al., 2006; Durkin et al., 2008b; Orr and Lanier, 2010). In a seminal report by Phillips et al. (1992), NK cells were found in human fetal liver as early as 6 weeks and in fetal spleen by 15 weeks of gestation. Similar to observations in fetal mice, these early human fetal liver NK cells were found to express high levels of the class Ib-specific CD94/NKG2 receptors. However, an analysis for the expression of the class Ia-specific KIR receptors was not included. The *in vitro* study of differentiating CD34+ hematopoietic progenitors indicates that CD94/NKG2A expression precedes the expression of HLA-specific KIR receptors by human NK cells (Grzywacz et al., 2006). For this reason, we examined the expression of KIR receptors by early gestation human fetal NK cells and found that small subsets of human fetal NK cells express adult levels of KIR receptor by 10 weeks of gestation with more appreciable levels identified by 14 weeks gestation (Alhajjat et al., 2010). A subsequent report by Ivarsson et al. (2013), demonstrated paradoxical hyporesponsivness to KIR-specific stimulation of second trimester human fetal NK cells. Hence, although early gestation human fetal NK cells possess the necessary machinery for allorecognition, a confirmation of their capacity for allospecific cytotoxicity requires future study.

## Closing Remarks

Successful engraftment in IUHCT likely requires tolerance in all components of the host immune system. Our studies in the murine model have indicated that a minimum level of circulating ligand is necessary to induce and maintain tolerance. This level seems to be higher for NK cells than the level required for other components of the immune system. Thus, the chimerism threshold might represent the minimum qualification in the education of the developing NK cells and NK cell tolerance as the final endorsement of donor recognition. The study of NK cell repertoire formation, maturity, and trogocytosis in prenatal transplantation will not only facilitate understanding of the NK cell barrier, but will also contribute to the overall understanding of NK cell biology.

## Conflict of Interest Statement

The authors declare that the research was conducted in the absence of any commercial or financial relationships that could be construed as a potential conflict of interest.
